# Supplementary material for: Investigating effect modification between childhood maltreatment and genetic risk for cardiovascular disease in the UK Biobank
Source: PLoS One. 2023 May 4;18(5):e0285258. doi: 10.1371/journal.pone.0285258 (PMC10159177; doi:10.1371/journal.pone.0285258)
Supplement: S1 File — (DOCX) [file pone.0285258.s002.docx]

# Investigating effect modification between childhood maltreatment and genetic risk for cardiovascular disease in the UK Biobank

Helena Urquijo^1,2*^, Ana Gonçalves Soares^1,2^, Abigail Fraser^1,2^, Laura D Howe^1,2^, Alice R Carter^1,2^

^1^ Medical Research Council Integrative Epidemiology Unit, University of Bristol

^2^ Population Health Sciences, Bristol Medical School, University of Bristol

* Corresponding author

E-mail: helena.urquijo@bristol.ac.uk (HU)

# Supplementary Material

[Supplementary Methods: Polygenic scores (PGS) 4](#_Toc124852134)

[Exclusion criteria 4](#_Toc124852135)

[Derivation of PGSs 4](#_Toc124852136)

[BMI 4](#_Toc124852137)

[Alcohol consumption (drinks per week) 4](#_Toc124852138)

[LDL cholesterol 4](#_Toc124852139)

[Lifetime smoking 5](#_Toc124852140)

[Systolic blood pressure 5](#_Toc124852141)

[Atrial Fibrillation 5](#_Toc124852142)

[Type 2 Diabetes 5](#_Toc124852143)

[Coronary Heart Disease 5](#_Toc124852144)

[Stroke 6](#_Toc124852145)

[Table S1 – Questions used to determine exposure to childhood maltreatment on mental health questionnaire. 7](#_Toc124852146)

[Table S2 – Evidence supporting a causal role for the considered risk factors on cardiovascular disease risk 7](#_Toc124852147)

[Table S3 – ICD codes for the included cardiovascular diseases/risk factors. 7](#_Toc124852148)

[Table S4 – Variance explained by each PGS according to the P value threshold. 9](#_Toc124852149)

[Table S5 – Descriptive statistics of study sample and entire Biobank sample (excluding withdrawn participants). 10](#_Toc124852150)

[Table S6 – Association of childhood maltreatment score with cardiovascular risk factors and disease. Analyses were adjusted for age and sex. 11](#_Toc124852151)

[Table S7 – Effect modification of genetic predisposition to cardiovascular risk factors and disease by childhood maltreatment score on the additive scale at P<5x10^-8^. 12](#_Toc124852152)

[Table S8 – Effect modification of genetic predisposition to cardiovascular risk factors and disease by childhood maltreatment score on the multiplicative scale at P<5x10^-8^. 13](#_Toc124852153)

[Table S9 – Sex-stratified effect modification of childhood maltreatment score on genetic predisposition to cardiovascular risk factors and disease on the additive scale at P<5x10^-8^. 14](#_Toc124852155)

[Table S10 – Sex-stratified effect modification of childhood maltreatment score on genetic predisposition to cardiovascular risk factors and disease on the multiplicative scale at P<5x10^-8^. 15](#_Toc124852157)

[Table S11 – Effect modification of genetic predisposition to cardiovascular risk factors and disease by childhood maltreatment score on the additive and multiplicative scale at P<0.05. 16](#_Toc124852159)

[Table S12 – Effect modification of genetic predisposition to cardiovascular risk factors and disease by childhood maltreatment score on the additive and multiplicative scale at P<0.5. 18](#_Toc124852161)

[Table S13 – Effect modification of genetic predisposition to cardiovascular risk factors and disease by childhood maltreatment score on the additive and multiplicative scale at P<5x10^-8^, adjusted for covariate interactions. 19](#_Toc124852163)

[Table S14 – Effect modification of genetic predisposition to cardiovascular risk factors and disease by exposure to childhood maltreatment on the additive and multiplicative scale at P<5x10^-8^, adjusted for childhood SEP. 20](#_Toc124852165)

[References 21](#_Toc124852167)

# Supplementary Methods: Polygenic scores (PGS)

## Exclusion criteria

Quality control of UK Biobank genetic data was performed in accordance with the MRC-IEU Quality Control pipeline, as previously described [[1]]. In brief, participants with sex mismatch (comparing genetic sex with reported sex) and those with sex chromosome aneuploidy were excluded from the study. Participants of White British ancestry were those who self-reported as “White British” and supported by genetic principal component (PC) analysis. Related participants were excluded according to kinship coefficients provided by the UK Biobank until no related pairs remained [[1]].

## Derivation of PGSs

Summary statistics were downloaded from MR-Base [2] using the R statistical package, including rs IDs, effect estimates, effect alleles and relevant study information. The 1000 Genomes Project was used to find proxy SNPs in LD (R^2^ above 0.8) with SNPs not found in UK Biobank. The SNPs from MR-Base were harmonised with the SNPs from UK Biobank, aligning the effect estimates and alleles. SNPs were clumped using the European subsample of the 1000 genomes project, with R^2^ < 0.001 and a window of 10,000 kb. PGS were calculated by multiplying the number of effect alleles for each participant in UK Biobank by the effect estimate of the SNP, then summing across all SNPs associated with each trait.

Suitable summary statistics were not available for SBP and lifetime smoking, thus a split sample GWAS of the UK Biobank was performed to generate PGSs. Briefly, the participants of the study were randomly allocated to one of two samples and a GWAS was performed on each of the samples. Summary statistics of each sample were then used to calculate PGSs for the other sample, avoiding sample overlap.[3] This method has been described in detail previously.[4]

## BMI

BMI (kg/m^2^) was calculated using the height and weight measurements collected at baseline.

We used summary statistics from the Genetic Investigation of Anthropometric Traits (GIANT) Consortium BMI GWAS on individuals of European ancestry (n=339 224),[5] the most recent BMI GWAS without UK Biobank participants to avoid sample overlap.

## Alcohol consumption (drinks per week)

Alcohol consumption was self-reported by participants at baseline at the assessment centres. Participants were asked to report their current drinking behaviours with the options daily or almost daily, three or four times a week, once or twice a week, one to three times a month, special occasions only, never or prefer not to say. Participants with an intake of once or twice weekly and over were asked to report their weekly intake of a number of alcoholic beverages; red wine, white wine, champagne, beer, cider, spirits, fortified wine. These were summed to create a variable reflecting the number of drinks per week, in which those which reported to drink one to three times a month or less were assumed to have an intake of 0 drinks per week. This variable has been previously described elsewhere.[6]

Summary statistics from the GWAS and Sequencing Consortium of Alcohol and Nicotine use (GSCAN) GWAS of drinks per week, as previously described,[7] were employed to generate the PGSs. UK Biobank participants were excluded to avoid sample overlap.

## LDL cholesterol

LDL cholesterol was measured from plasma samples collected from participants by enzymatic protective selection analysis on a Beckman Coulter AU5800. Measurements were excluded by UK Biobank if they did not pass the UK Biobank’s quality control checks after an inadvertent dilution of samples.[8]

Summary statistics were taken from the Global Lipids Genetics Consortium LDL cholesterol GWAS of individuals of European ancestry (n=188,577).[9]

## Lifetime smoking

Smoking behaviour was self-reported by participants at baseline at the assessment centres. Participants were asked about smoking status (current, former or never), age of initiation, and cigarettes smoked per day. A variable reflecting lifetime smoking behaviour was generated by combining the smoking measures into an index and a half-life constant reflecting the exponential decrease in effect of smoking at a given time on health. A detailed overview of the construction of this variable can be found elsewhere. [10]

For the GWAS, estimates from the statistical analyses using the two samples were meta-analysed by using the *metan* command on Stata.

## Systolic blood pressure

Participant systolic blood pressure measurements were taken at baseline while attending assessment centres using an Omron HEM-7105IT blood pressure monitor. The mean of two resting blood pressure measurements was used.

For the GWAS summary statistics, estimates from the statistical analyses using the two samples were meta-analysed by using the *metan* command on Stata.

## Atrial Fibrillation

Atrial fibrillation cases were determined through mortality data from the UK Biobank and linked hospital episode statistics and Scottish morbidity and mortality records, referred to jointly as hospital inpatient records. Cases were identified according to the specified ICD9 and ICD10 disease code as shown in Table S3. Date of diagnosis and date of attendance of assessment centre were compared to distinguish prevalent from incident cases.

Summary statistics were obtained from an Atrial Fibrillation GWAS of individuals of European ancestry (n=59 133, of which 6 707 with atrial fibrillation).[11]

## Type 2 Diabetes

T2DM cases were determined through mortality data from the UK Biobank and linked hospital inpatient records. Date of diagnosis and date of attendance of assessment centre were compared to distinguish prevalent from incident cases. Given the relatively low proportion of hospitalisation episodes attributed to T2DM alone, self-reported cases to UK Biobank nurses at an assessment centre were included. Cases reported at baseline were identified as prevalent if reported at baseline and as incident if reported at a follow-up visit to the assessment centre, though only a proportion of participants attend these. These self-reported variables do not distinguish between type 1 and type 2 diabetes, yet most of these cases are expected to be of the latter.

Summary statistics were taken from the ﻿DIAbetes Genetics Replication And Meta-analysis (DIAGRAM) Consortium GWAS of individuals of European ancestry (n=﻿159,234 of which 26,676 with T2DM).[12]

## Coronary Heart Disease

CHD cases were determined through mortality data from the UK Biobank and linked hospital inpatient records. Cases were identified according to the specified ICD9 and ICD10 disease codes as shown in Table S3, spanning subtypes such as acute myocardial infarction and angina pectoris. Date of diagnosis and date of attendance of assessment centre were compared to distinguish prevalent from incident cases.

Summary statistics were taken from the most recent CHD GWAS without UK Biobank participants. The GWAS included individuals of predominantly (77%) European ancestry (n=184,305, of which 60,801 with CHD). [13]

## Stroke

Stroke cases were determined through mortality data from the UK Biobank and linked hospital inpatient records. Cases were identified according to the specified ICD9 and ICD10 disease code as shown in Table S3. Date of diagnosis and date of attendance of assessment centre were compared to distinguish prevalent from incident cases.

Summary statistics were obtained from the MEGASTROKE Consortium GWAS including individuals European ancestry (n=446,696, of which cases = 40,585).[14]

# Table S1 – Questions used to determine exposure to childhood maltreatment on mental health questionnaire.

| **Question: “When I was a child…”** | **Type of maltreatment** | **Cut-off ^a^** |
| --- | --- | --- |
| “I felt loved” | Emotional neglect | 2 or lower |
| “People in my family hit me so hard that it left me with bruises or marks” | Physical abuse | 1 or higher |
| “I felt that someone in my family hated me” | Emotional abuse | 1 or higher |
| “Someone molested me (sexually)” | Sexual abuse | 1 or higher |
| “There was someone to take me to the doctor if I needed it” | Physical neglect | 3 or lower |

^a^According to the following answers: 0: never true; 1: rarely true; 2: sometimes true; 3: often true; 4: very often true.

# Table S2 – Evidence supporting a causal role for the considered risk factors on cardiovascular disease risk

| **Risk factor** | **Study** | **Study design** |
| --- | --- | --- |
| Alcohol (drinks per week) | Millwood *et al,* 2019 [15] | Mendelian randomization study |
| BMI | Carter *et al,* 2019 [4]; Larsson *et al,* 2020 [16] | Mendelian randomization study; Mendelian randomization study |
| Low density lipoprotein cholesterol | Ference et al, 2017 [17]; Cheung et al, 2004 [18] | Mendelian randomization and clinical study; randomized controlled trial |
| Smoking (lifetime behaviour) | Larsson *et al,* 2020 [19] | Mendelian randomization study |
| Systolic blood pressure | Carter *et al,* 2019 [4]; Ettehad *et al,* 2016 [20] | Mendelian randomization study; systematic review including randomized controlled trial |
| Type 2 diabetes | Ahmad *et al,* 2015 [21] | Mendelian randomization study |

# Table S3 – ICD codes for the included cardiovascular diseases/risk factors.

| **Diagnosis** | **ICD9** | **ICD10** |
| --- | --- | --- |
| Atrial Fibrillation | 427.31 | I48 |
| Type 2 diabetes | 250.00, 250.02, 250.10, 250.12, 250.20, 250.22, 250.30, 250.32, 250.40, 250.42, 250.50, 250.52, 250.60, 250.62, 250.70, 250.72, 250.80, 250.82, 250.90, 250.92 | E11 |
| Coronary Heart Disease | 410.0-414.9 | I20-I25 |
| Stroke | 430.0-438.9 | G45, I60-I64 |

# Table S4 – Variance explained by each PGS according to the P value threshold.

| **Risk factor/disease GWAS** | **P<5×10^-8^** | | **P<0.05** | | **P<0.5** | |
| --- | --- | --- | --- | --- | --- | --- |
|  | **# of SNPs** | **R^2^** | **# of SNPs** | **R^2^** | **# of SNPs** | **R^2^** |
| BMI | 127 | 0.02983 | 20 542 | 0.06512 | 139 582 | 0.06868 |
| Drinks per week | 14 | 0.07834 | 72 962 | 0.08039 | 449 080 | 0.08019 |
| LDL cholesterol | 398 | 0.07026 | 13 337 | 0.02450 | 23 724 | 0.02288 |
| Lifetime smoking (sample 1) | 23 | 0.00621 | 67 741 | 0.01571 | 391 104 | 0.01606 |
| Lifetime smoking (sample 2) | 21 | 0.00641 | 66 909 | 0.01516 | 390 557 | 0.01559 |
| Systolic blood pressure (sample 1) | 126 | 0.15067 | 77 709 | 0.17562 | 373 402 | 0.17260 |
| Systolic blood pressure (sample 2) | 112 | 0.14150 | 76 557 | 0.16176 | 372 715 | 0.16011 |
| Atrial Fibrillation | 3431 | 0.09755 | 60 738 | 0.29849 | 361 969 | 0.38717 |
| Type 2 Diabetes | 18 | 0.02916 | 5 137 | 0.02422 | 134 673 | 0.02300 |
| Coronary Heart Disease | 75 | 0.06554 | 49 098 | 0.06453 | 345 040 | 0.06356 |
| Stroke | 11 | 0.03919 | 63 025 | 0.03948 | 373 240 | 0.03919 |

# Table S5 – Descriptive statistics of study sample and entire Biobank sample (excluding withdrawn participants).

| **Variable** | **Analysis sample N = 100 833** | | | **Full UK Biobank N = 502 493** | | |
| --- | --- | --- | --- | --- | --- | --- |
| **Continuous** | **N** | **Mean (SD)** | | **N** | **Mean (SD)** | |
| Age | 100 833 | 55.88 (7.67) | | 502 493 | 56.53 (8.10) | |
| BMI | 100 659 | 26.57 (4.38) | | 499 389 | 27.43 (4.80) | |
| Drinks per week | 100 529 | 8.38 (8.66) | | 498 251 | 7.79 (9.05) | |
| LDL cholesterol | 95 809 | 3.64 (0.82) | | 468 696 | 3.56 (0.87) | |
| Lifetime smoking | 95 845 | 0.25 (0.55) | | 318 071 | 0.34 (0.67) | |
| Systolic blood pressure | 93 001 | 136.37 (18.08) | | 456 954 | 137.78 (18.63) | |
| Number of siblings | 99 629 | 1.78 (1.47) | | 492 858 | 2.23 (2.00) | |
| **Categorical** | **N** | **Frequency (%)** | | **N** | **Frequency (%)** | |
| Sex | 100833 | Female | 57 641 (57) | 502493 | Female | 273 378 (54) |
| Maternal smoking | 88907 | No | 63 353 (71) | 434357 | No | 307 274 (71) |
| Atrial fibrillation (incident) | 100833 | Control | 98 735 (97) | 496107 | Control | 480 339 (97) |
|  |  | Case | 2 098 (2) |  | Case | 15 768 (3) |
| Type 2 Diabetes (incident) | 100833 | Control | 99 449 (99) | 493064 | Control | 47 2429 (96) |
|  |  | Case | 1 384 (1) |  | Case | 20 635 (4) |
| Coronary Heart Disease (incident) | 100833 | Control | 97 801 (97) | 481868 | Control | 45 9019 (95) |
|  |  | Case | 3 032 (3) |  | Case | 22 849 (5) |
| Stroke (incident) | 100833 | Control | 99 838 (99) | 497488 | Control | 48 7421 (98) |
|  |  | Case | 995 (1) |  | Case | 10 067 (2) |
| Physical abuse | 100833 | Yes | 17 530 (17) | 156982 | Yes | 29 793 (19) |
| Emotional abuse |  |  | 14 535 (14) | 156886 |  | 24 544 (16) |
| Sexual abuse |  |  | 8 191 (8) | 155488 |  | 13 646 (9) |
| Physical neglect |  |  | 15 286 (15) | 156266 |  | 25 723 (16) |
| Emotional neglect |  |  | 21 189 (21) | 156712 |  | 35 290 (23) |
| Childhood maltreatment score | 100833 | 0 | 55 910 (55) | 153625 | 0 | 82 398 (54) |
|  |  | 1 | 25 570 (25) |  | 1 | 39 207 (26) |
|  |  | 2 | 10 846 (11) |  | 2 | 17 482 (11) |
|  |  | 3 | 5 299 (5) |  | 3 | 8 801 (6) |
|  |  | 4 | 2 468 (2) |  | 4 | 4 390 (3) |
|  |  | 5 | 740 (1) |  | 5 | 1 347 (1) |

For maltreatment types, exposure is determined according to cut-off criteria described in Table S1.

# Table S6 – Association of childhood maltreatment score with cardiovascular risk factors and disease. Analyses were adjusted for age and sex.

| **Continuous trait** | **Mean difference in SD of phenotypic trait per unit increase in maltreatment score (95% CI)** |
| --- | --- |
| BMI | 0.068 (0.062, 0.073) |
| Drinks per week | 0.002 (-0.003, 0.008) |
| LDL cholesterol | 0.003 (-0.003, 0.009) |
| Lifetime smoking | 0.101 (0.095, 0.107) |
| Systolic blood pressure | -0.011 (-0.017, -0.005) |
| **Binary trait** | **Odds ratio per unit increase in maltreatment score (95% CI)** |
| Atrial Fibrillation | 1.04 (0.99, 1.08) |
| Type 2 Diabetes | 1.19 (1.14, 1.25) |
| Coronary Heart Disease | 1.13 (1.10, 1.17) |
| Stroke | 1.06 (1.00, 1.12) |

| **Continuous**  **trait** | **N** | | **Additive scale** | | | |
| --- | --- | --- | --- | --- | --- | --- |
|  |  |  | **Mean difference in SD of trait per SD increase of PGS (95% CI)** | **Mean difference in SD of trait per unit increase of maltreatment score (95% CI)** | **Interaction coefficient** | **P value for interaction** |
| BMI | 100659 | | 0.12 (0.11, 0.13) | 0.065 (0.060, 0.071) | 0.009 (0.003, 0.014) | 0.003 |
| Drinks per week | 100529 | | 0.04 (0.04, 0.05) | 0.001 (-0.004, 0.007) | 0.002 (-0.004, 0.007) | 0.598 |
| LDL cholesterol | 95809 | | 0.23 (0.22, 0.23) | 0.003 (-0.002, 0.009) | 0.003 (-0.002, 0.009) | 0.238 |
| Lifetime smoking | 95845 | | 0.04 (0.03, 0.04) | 0.100 (0.094, 0.105) | 0.003 (-0.003, 0.008) | 0.395 |
| Systolic blood pressure | 93001 | | 0.09 (0.08, 0.10) | -0.011 (-0.016, -0.005) | -0.002 (-0.008, 0.004) | 0.490 |
| **Binary trait** | **N = 100833** | | **Risk difference of trait per SD increase of PGS (95% CI)** | **Risk difference of trait per SD increase of maltreatment score (95% CI)** | **Interaction coefficient** | **P value for interaction** |
| Atrial fibrillation | Control | 98735 | 0.011 (0.010, 0.012) | 0.001 (0.000, 0.002) | 4x10^-4^ (-4x10^-4^, 1x10^-3^) | 0.322 |
|  | Case | 2098 |  |  |  |  |
| Type 2 diabetes | Control | 99449 | 0.003 (0.003, 0.004) | 0.003 (0.002, 0.003) | 4x10^-4^ (-2x10^-4^, 1x10^-3^) | 0.196 |
|  | Case | 1384 |  |  |  |  |
| Coronary heart disease | Control | 97801 | 0.007 (0.006, 0.008) | 0.004 (0.003, 0.005) | 3x10^-4^ (-1 x10^-3^, 1x10^-3^) | 0.611 |
|  | Case | 3032 |  |  |  |  |
| Stroke | Control | 99838 | 1x10^-4^ (-5x10^-4^, 8x10^-4^) | 0.001 (-0.000, 0.001) | 1x10^-5^ (-5x10^-4^, 6x10^-4^) | 0.973 |
|  | Case | 995 |  |  |  |  |

# Table S7 – Effect modification of genetic predisposition to cardiovascular risk factors and disease by childhood maltreatment score on the additive scale at P<5x10^-8^. Analyses were adjusted for age, sex and 40 genetic principal components.

# Table S8 – Effect modification of genetic predisposition to cardiovascular risk factors and disease by childhood maltreatment score on the multiplicative scale at P<5x10^-8^.

| **Continuous trait** | **N** | | **Multiplicative scale** | | | |
| --- | --- | --- | --- | --- | --- | --- |
|  |  |  | **Mean difference in SD of log trait per SD increase of PGS (95% CI)** | **Mean difference in log SD of trait per unit increase of maltreatment score (95% CI)** | **Interaction coefficient** | **P value for interaction** |
| BMI | 100659 | | 0.12 (0.11, 0.13) | 0.065 (0.059, 0.070) | 0.007 (0.001, 0.013) | 0.015 |
| Drinks per week | 100529 | | 0.05 (0.04, 0.06) | -0.028 (-0.033, -0.022) | 0.002 (-0.004, 0.008) | 0.496 |
| LDL cholesterol | 95809 | | 0.23 (0.22, 0.23) | 0.002 (-0.003, 0.008) | 0.004 (-0.002, 0.010) | 0.164 |
| Lifetime smoking | 95845 | | 0.04 (0.03, 0.04) | 0.103 (0.097, 0.109) | 0.002 (-0.003, 0.008) | 0.429 |
| Systolic blood pressure | 93001 | | 0.09 (0.09, 0.10) | -0.011 (-0.017, -0.006) | -0.002 (-0.007, 0.004) | 0.506 |
| **Binary trait** | **N** | | **OR of trait per SD increase of PGS (95% CI)** | **OR of trait per unit increase of maltreatment score (95% CI)** | **Interaction coefficient** | **P value for interaction** |
| Atrial fibrillation | Control | 98735 | 1.69 (1.62, 1.76) | 1.03 (0.98, 1.08) | 1.02 (0.98, 1.06) | 0.312 |
|  | Case | 2098 |  |  |  |  |
| Type 2 diabetes | Control | 99449 | 1.29 (1.22, 1.35) | 1.20 (1.14, 1.25) | 0.99 (0.95, 1.04) | 0.743 |
|  | Case | 1384 |  |  |  |  |
| Coronary heart disease | Control | 97801 | 1.28 (1.23, 1.32) | 1.14 (1.10, 1.18) | 0.99 (0.96, 1.02) | 0.615 |
|  | Case | 3032 |  |  |  |  |
| Stroke | Control | 99838 | 1.02 (0.95, 1.08) | 1.06 (1.00, 1.12) | 1.00 (0.94, 1.06) | 0.990 |
|  | Case | 995 |  |  |  |  |

# Analyses were adjusted for age, sex and 40 genetic principal components.

# Table S9 – Sex-stratified effect modification of childhood maltreatment score on genetic predisposition to cardiovascular risk factors and disease on the additive scale at P<5x10^-8^.

# Analyses were adjusted for age, sex and 40 genetic principal components. BMI=body mass index; LDL=low-density lipoprotein; PGS=polygenic score.

| **Continuous trait** | **N** | | **Additive scale** | | | | | | | | |
| --- | --- | --- | --- | --- | --- | --- | --- | --- | --- | --- | --- |
|  |  |  | **Sex** | **N** | **Mean difference in SD of trait per SD increase of PGS (95% CI)** | **Mean difference in SD of trait per unit increase of maltreatment score (95% CI)** | **Interaction coefficient** | | **P Value for interaction** | | **P value interaction by sex** |
| BMI | 100659 | | Female | 57540 | 0.13 (0.12, 0.14) | 0.067 (0.059, 0.074) | 0.007 (-0.001, 0.015) | | 0.071 | | 0.596 |
|  |  |  | Male | 43119 | 0.11 (0.11, 0.12) | 0.064 (0.056, 0.072) | 0.010 (0.002, 0.019) | | 0.016 | |  |
| Drinks per week | 100529 | | Female | 57541 | 0.04 (0.03, 0.05) | -0.002 (-0.008, 0.004) | 3E-04 (-0.006, 0.006) | | 0.926 | | 0.503 |
|  |  |  | Male | 42988 | 0.05 (0.04, 0.06) | 0.005 (-0.005, 0.016) | 0.004 (-0.006, 0.015) | | 0.429 | |  |
| LDL cholesterol | 95809 | | Female | 54698 | 0.24 (0.23, 0.25) | 0.009 (0.002, 0.016) | 0.002 (-0.005, 0.009) | | 0.604 | | 0.506 |
|  |  |  | Male | 41111 | 0.21 (0.20, 0.22) | -0.002 (-0.011, 0.007) | 0.004 (-0.005, 0.013) | | 0.406 | |  |
| Lifetime smoking | 95845 | | Female | 54768 | 0.03 (0.02, 0.04) | 0.095 (0.088, 0.102) | 0.001 (-0.006, 0.008) | | 0.735 | | 0.440 |
|  |  |  | Male | 41077 | 0.04 (0.03, 0.05) | 0.107 (0.097, 0.117) | 0.006 (-0.004, 0.016) | | 0.258 | |  |
| Systolic blood pressure | 93001 | | Female | 53143 | 0.10 (0.09, 0.11) | -0.010 (-0.018, -0.003) | -0.004 (-0.011, 0.004) | | 0.320 | | 0.572 |
|  |  |  | Male | 39858 | 0.09 (0.08, 0.10) | -0.009 (-0.018, 0.000) | -2X10^-5^ (-9x10^-3^, 9x10^-3^) | | 0.972 | |  |
| **Binary trait** | **N = 100 833** | | **Sex**  **Female N= 57641**  **Male N = 43192** | | **Risk difference of trait per SD increase of PGS (95% CI)** | **Risk difference of trait per SD increase of maltreatment score (95% CI)** | **Interaction coefficient** | | **P value for interaction** | | **P value interaction by sex** |
| Atrial fibrillation | Control | 98735 | Female | | 0.007 (0.006, 0.008) | 2x10^-4^ (-6x10^-4^, 0.001) | 4x10^-4^ (-4x10^-4^, 0.001) | 0.350 | | 0.432 | |
|  | Case | 2098 | Male | | 0.017 (0.016, 0.019) | 0.002 (0.000, 0.003) | 0.001 (-0.001, 0.003) | 0.226 | |  |  |
| Type 2 diabetes | Control | 99449 | Female | | 0.003 (0.002, 0.004) | 0.002 (0.001, 0.003) | 0.001 (0.000, 0.001) | 0.046 | | 0.349 | |
|  | Case | 1384 | Male | | 0.004 (0.003, 0.005) | 0.004 (0.003, 0.005) | 6x10-5 (-0.001, 0.001) | 0.926 | |  |  |
| Coronary heart disease | Control | 97801 | Female | | 0.004 (0.003, 0.005) | 0.003 (0.002, 0.004) | -3x10^-4^ (-1x10-3, 1x10-3) | 0.527 | | 0.065 | |
|  | Case | 3032 | Male | | 0.011 (0.009, 0.013) | 0.004 (0.002, 0.006) | 0.002 (-4x10^-4^, 0.004) | 0.116 | |  |  |
| Stroke | Control | 99838 | Female | | 2x10^-4^ (-5x10^-4^, 9x10^-4^) | 0.001 (0.000, 0.001) | 5x10^-4^ (-1x10^-4^, 1x10^-3^) | 0.106 | | 0.020 | |
|  | Case | 995 | Male | | 1x10^-4^ (-1x10^-3^, 1x10^-3^) | 2x10^-4^ (-9x10^-4^, 1x10^-3^) | -1x10^-4^ (-2x10^-3^, 2x10^-4^) | 0.118 | |  |  |

# Table S10 – Sex-stratified effect modification of childhood maltreatment score on genetic predisposition to cardiovascular risk factors and disease on the multiplicative scale at P<5x10^-8^.

| **Continuous trait** | **N** | | **Sex** | **N** | **Multiplicative scale** | | | | |
| --- | --- | --- | --- | --- | --- | --- | --- | --- | --- |
|  |  |  |  |  | **Mean difference in SD of log trait per SD increase of PGS (95% CI)** | **Mean difference in log SD of trait per unit increase of maltreatment score (95% CI)** | **Interaction coefficient** | **P value for Interaction** | **P value interaction by sex** |
| BMI | 100659 | | Female | 57540 | 0.13 (0.12, 0.14) | 0.066 (0.059, 0.074) | 0.006 (-0.002, 0.013) | 0.145 | 0.635 |
|  |  |  | Male | 43119 | 0.11 (0.11, 0.12) | 0.062 (0.053, 0.070) | 0.009 (3x10-4, 0.017) | 0.042 |  |
| Drinks per week | 100529 | | Female | 57541 | 0.05 (0.05, 0.06) | -0.028 (-0.035, -0.021) | 0.001 (-0.006, 0.008) | 0.839 | 0.616 |
|  |  |  | Male | 42988 | 0.04 (0.03, 0.05) | -0.028 (-0.037, -0.019) | 0.004 (-0.005, 0.013) | 0.402 |  |
| LDL cholesterol | 95809 | | Female | 54698 | 0.24 (0.23, 0.25) | 0.008 (0.001, 0.015) | 0.003 (-0.004, 0.010) | 0.465 | 0.544 |
|  |  |  | Male | 41111 | 0.21 (0.20, 0.21) | -0.003 (-0.013, 0.006) | 0.004 (-0.005, 0.014) | 0.379 |  |
| Lifetime smoking | 95845 | | Female | 54768 | 0.03 (0.02, 0.04) | 0.099 (0.092, 0.106) | 0.002 (-0.005, 0.009) | 0.620 | 0.672 |
|  |  |  | Male | 41077 | 0.05 (0.04, 0.06) | 0.108 (0.098, 0.119) | 0.004 (-0.006, 0.015) | 0.396 |  |
| Systolic blood pressure | 93001 | | Female | 53143 | 0.10 (0.09, 0.11) | -0.011 (-0.018, -0.004) | -0.004 (-0.011, 0.003) | 0.280 | 0.477 |
|  |  |  | Male | 39858 | 0.09 (0.08, 0.09) | -0.009 (-0.018, -0.001) | 4x10-4 (-0.008, 0.009) | 0.934 |  |
| **Binary trait** | **N = 100 833** | | **Sex**  **Female N= 57641**  **Male N = 43192** | | **OR of trait per SD increase of PGS (95% CI)** | **OR of trait per unit increase of maltreatment score (95% CI)** | **Interaction coefficient** | **P value for Interaction** | **P value interaction by sex** |
| Atrial fibrillation | Control | 98735 | Female | | 1.61 (1.50, 1.72) | 1.00 (0.93, 1.07) | 1.03 (0.97, 1.10) | 0.291 | 0.683 |
|  | Case | 2098 | Male | | 1.74 (1.65, 1.84) | 1.05 (0.99, 1.12) | 1.01 (0.96, 1.07) | 0.588 |  |
| Type 2 diabetes | Control | 99449 | Female | | 1.34 (1.24, 1.46) | 1.18 (1.10, 1.26) | 1.01 (0.95, 1.08) | 0.651 | 0.324 |
|  | Case | 1384 | Male | | 1.25 (1.17, 1.33) | 1.22 (1.14, 1.29) | 0.97 (0.91, 1.03) | 0.341 |  |
| Coronary heart disease | Control | 97801 | Female | | 1.23 (1.16, 1.31) | 1.20 (1.14, 1.26) | 0.96 (0.92, 1.01) | 0.127 | 0.088 |
|  | Case | 3032 | Male | | 1.30 (1.24, 1.36) | 1.09 (1.04, 1.14) | 1.02 (0.97, 1.07) | 0.401 |  |
| Stroke | Control | 99838 | Female | | 1.03 (0.93, 1.13) | 1.09 (1.01, 1.19) | 1.06 (0.98, 1.15) | 0.136 | 0.029 |
|  | Case | 995 | Male | | 1.01 (0.93, 1.10) | 1.01 (0.93, 1.10) | 0.94 (0.86, 1.02) | 0.123 |  |

# Analyses were adjusted for age, sex and 40 genetic principal components. BMI=body mass index; LDL=low-density lipoprotein; PGS=polygenic score.

# Table S11 – Effect modification of genetic predisposition to cardiovascular risk factors and disease by childhood maltreatment score on the additive and multiplicative scale at P<0.05.

# Analyses were adjusted for age, sex and 40 genetic principal components. BMI=body mass index; LDL=low-density lipoprotein; PGS=polygenic score.

|  | **P<0.05** | | | | | | | |
| --- | --- | --- | --- | --- | --- | --- | --- | --- |
| **Continuous Trait** | **Additive scale** | | | | **Multiplicative scale** | | | |
|  | **Mean difference in SD of trait per SD increase of PGS (95% CI)** | **Mean difference in SD of trait per unit increase of maltreatment score (95% CI)** | **Interaction coefficient** | **P value of interaction** | **Mean difference in SD of log trait per SD increase of PGS (95% CI)** | **Mean difference in log SD of trait per unit increase of maltreatment score (95% CI)** | **Interaction coefficient** | **P value of interaction** |
| BMI | 0.23 (0.22, 0.23) | 0.061 (0.056, 0.067) | 0.016 (0.010, 0.021) | 2x10^-8^ | 0.23 (0.22, 0.23) | 0.060 (0.055, 0.066) | 0.013 (0.008, 0.019) | 4x10^-6^ |
| Drinks per week | 0.06 (0.06, 0.07) | 0.001 (-0.005, 0.007) | -0.002 (-0.007, 0.004) | 0.465 | 0.06 (0.06, 0.07) | -0.028 (-0.033, -0.022) | -0.002 (-0.008, 0.004) | 0.480 |
| LDL cholesterol | 0.08 (0.07, 0.09) | 0.003 (-0.003, 0.009) | 3x10^-4^ (-6x10^-3^, 6x10^-3^) | 0.917 | 0.08 (0.07, 0.09) | 0.002 (-0.004, 0.008) | 2x10^-4^ (-6x10^-3^, 6x10^-3^) | 0.941 |
| Lifetime smoking | 0.12 (0.11, 0.13) | 0.206 (0.143, 0.269) | 0.017 (0.009, 0.024) | 7x10^-6^ | 0.12 (0.12, 0.13) | 0.215 (0.152, 0.278) | 0.014 (0.007, 0.022) | 1x10^-4^ |
| Systolic blood pressure | 0.21 (0.20, 0.22) | 0.043 (-0.017, 0.101) | -9x10^-5^ (-7x10^-3^, 6x10^-3^) | 0.978 | 0.21 (0.21, 0.22) | 0.037 (-0.022, 0.095) | 0.001 (-0.006, 0.007) | 0.223 |
| **Binary Trait** | **Risk difference of trait per SD increase of PGS (95% CI)** | **Risk difference of trait per SD increase of maltreatment score (95% CI)** | **Interaction coefficient** | **P value of interaction** | **OR of trait per SD increase of PGS (95% CI)** | **OR of trait per unit increase of maltreatment score (95% CI)** | **Interaction coefficient** | **P value of interaction** |
| Atrial fibrillation | 0.032 (0.031, 0.033) | 0.001 (0.000, 0.001) | -6x10^-4^ (-1x10^-4^, 2x10^-4^) | 0.122 | 4.56 (4.35, 4.79) | 1.06 (0.98, 1.14) | 0.98 (0.93, 1.02) | 0.365 |
|  |  |  |  |  |  |  |  |  |
| Type 2 diabetes | 0.002 (0.001, 0.002) | 0.003 (0.002, 0.003) | 2x10^-4^ (-5x10^-4^, 9x10^-4^) | 0.575 | 1.12 (1.06, 1.18) | 1.20 (1.14, 1.25) | 1.00 (0.95, 1.04) | 0.867 |
|  |  |  |  |  |  |  |  |  |
| Coronary heart disease | 0.006 (0.005, 0.007) | 0.004 (0.003, 0.004) | -3x10^-5^ (-1x10^-3^, 1x10^-3^) | 0.954 | 1.25 (1.20, 1.30) | 1.13 (1.10, 1.17) | 0.98 (0.95, 1.02) | 0.367 |
|  |  |  |  |  |  |  |  |  |
| Stroke | 0.001 (0.000, 0.001) | 0.001 (0.000, 0.001) | -1x10-5 (-6x10-4, 6x10^-4^) | 0.960 | 1.06 (1.00, 1.13) | 1.06 (0.99, 1.12) | 1.00 (0.94, 1.06) | 0.953 |
|  |  |  |  |  |  |  |  |  |

# Table S12 – Effect modification of genetic predisposition to cardiovascular risk factors and disease by childhood maltreatment score on the additive and multiplicative scale at P<0.5.

|  | **P<0.5** | | | | | | | |
| --- | --- | --- | --- | --- | --- | --- | --- | --- |
| **Continuous Trait** | **Additive scale** | | | | **Multiplicative scale** | | | |
|  | **Mean difference in SD of trait per SD increase of PGS (95% CI)** | **Mean difference in SD of trait per unit increase of maltreatment score (95% CI)** | **Interaction coefficient** | **P value of interaction** | **Mean difference in SD of log trait per SD increase of PGS (95% CI)** | **Mean difference in log SD of trait per unit increase of maltreatment score (95% CI)** | **Interaction coefficient** | **P value of interaction** |
| BMI | 0.23 (0.23, 0.24) | 0.061 (0.055, 0.066) | 0.015 (0.009, 0.020) | 1x10^-7^ | 0.24 (0.23, 0.24) | 0.060 (0.054, 0.066) | 0.011 (0.007, 0.017) | 2x10^-5^ |
| Drinks per week | 0.06 (0.06, 0.07) | 0.001 (-0.005, 0.007) | -0.002 (-0.008, 0.003) | 0.467 | 0.06 (0.06, 0.07) | -0.028 (-0.033, -0.022) | -0.002 (-0.008, 0.003) | 0.397 |
| LDL cholesterol | 0.07 (0.06, 0.08) | 0.003 (-0.003, 0.009) | 0.003 (-0.003, 0.009) | 0.276 | 0.07 (0.06, 0.07) | 0.002 (-0.004, 0.008) | 0.003 (-0.003, 0.008) | 0.377 |
| Lifetime smoking | 0.14 (0.13, 0.14) | 0.201 (0.138, 0.264) | 0.022 (0.014, 0.030) | 3x10^-8^ | 0.14 (0.13, 0.15) | 0.210 (0.147, 0.273) | 0.020 (0.012, 0.028) | 9x10^-7^ |
| Systolic blood pressure | 0.22 (0.21, 0.23) | 0.036 (-0.023, 0.096) | 0.002 (-0.005, 0.009) | 0.574 | 0.22 (0.22, 0.23) | 0.031 (-0.028, 0.090) | 0.003 (-0.004, 0.010) | 0.413 |
| **Binary Trait** | **Risk difference of trait per SD increase of PGS (95% CI)** | **Risk difference of trait per SD increase of maltreatment score (95% CI)** | **Interaction coefficient** | **P value of interaction** | **OR of trait per SD increase of PGS (95% CI)** | **OR of trait per unit increase of maltreatment score (95% CI)** | **Interaction coefficient** | **P value of interaction** |
| Atrial fibrillation | 0.040 (0.039, 0.041) | 0.001 (-0.000, 0.001) | -0.001 (-0.002, 0.000) | 0.066 | 5.37 (5.11, 5.64) | 1.06 (0.97, 1.15) | 0.98 (0.94, 1.03) | 0.432 |
|  |  |  |  |  |  |  |  |  |
| Type 2 diabetes | 2x10^-4^ (-5x10^-4^, 9x10^-4^) | 0.003 (0.002, 0.003) | 2x10^-4^ (-5x10^-4^, 9x10^-4^) | 0.535 | 1.02 (0.96, 1.07) | 1.20 (1.14, 1.25) | 1.01 (0.97, 1.06) | 0.615 |
|  |  |  |  |  |  |  |  |  |
| Coronary heart disease | 0.006 (0.005, 0.007) | 0.004 (0.003, 0.005) | -1x10^-4^ (-1x10^-3^, 8x10^-4^) | 0.782 | 1.22 (1.18, 1.27) | 1.14 (1.10, 1.17) | 0.98 (0.95, 1.02) | 0.331 |
|  |  |  |  |  |  |  |  |  |
| Stroke | 1x10^-4^ (-5x10^-4^, 8x10^-4^) | 0.001 (0.000, 0.001) | 4x10^-5^ (-5x10^-4^, 6x10^-4^) | 0.901 | 1.02 (0.95, 1.08) | 1.06 (1.00, 1.12) | 1.01 (0.95, 1.07) | 0.865 |
|  |  |  |  |  |  |  |  |  |

# Analyses were adjusted for age, sex and 40 genetic principal components. BMI=body mass index; LDL=low-density lipoprotein; PGS=polygenic score.

|  | **Adjusted for covariate interactions, P<5x10^-8^** | | | | | | | |
| --- | --- | --- | --- | --- | --- | --- | --- | --- |
| **Continuous Trait** | **Additive scale** | | | | **Multiplicative scale** | | | |
|  | **Mean difference in SD of trait per SD increase of PGS (95% CI)** | **Mean difference in SD of trait per unit increase of maltreatment score (95% CI)** | **Interaction coefficient** | **P value of interaction** | **Mean difference in SD of log trait per SD increase of PGS (95% CI)** | **Mean difference in log SD of trait per unit increase of maltreatment score (95% CI)** | **Interaction coefficient** | **P value of interaction** |
| BMI | 0.12 (0.11, 0.13) | 0.099 (0.038, 0.160) | 0.007 (0.001, 0.012) | 0.015 | 0.12 (0.11, 0.13) | 0.094 (0.033, 0.155) | 0.006 (-0.000, 0.011) | 0.054 |
| Drinks per week | 0.04 (0.04, 0.05) | -0.004 (-0.063, 0.056) | 0.002 (-0.004, 0.007) | 0.543 | 0.05 (0.04, 0.06) | -0.068 (-0.129, -0.008) | 0.002 (-0.004, 0.007) | 0.598 |
| LDL cholesterol | 0.23 (0.22, 0.23) | 0.011 (-0.050, 0.072) | 0.002 (-0.004, 0.008) | 0.476 | 0.23 (0.22, 0.23) | 0.014 (-0.047, 0.076) | 0.002 (-0.004, 0.008) | 0.452 |
| Lifetime smoking | 0.04 (0.03, 0.04) | 0.215 (0.152, 0.279) | 0.002 (-0.003, 0.008) | 0.463 | 0.04 (0.03, 0.04) | 0.224 (0.161, 0.287) | 0.002 (-0.004, 0.008) | 0.515 |
| Systolic blood pressure | 0.09 (0.08, 0.10) | 0.039 (-0.021, 0.099) | -0.002 (-0.008, 0.004) | 0.395 | 0.09 (0.09, 0.10) | 0.034 (-0.026, 0.093) | -0.003 (-0.008, 0.003) | 0.366 |
| **Binary Trait** | **Risk difference of trait per SD increase of PGS (95% CI)** | **Risk difference of trait per SD increase of maltreatment score (95% CI)** | **Interaction coefficient** | **P value of interaction** | **OR of trait per SD increase of PGS (95% CI)** | **OR of trait per unit increase of maltreatment score (95% CI)** | **Interaction coefficient** | **P value of interaction** |
| Atrial fibrillation | 0.011 (0.010, 0.012) | -0.002 (-0.011, 0.006) | 0.001 (0.000, 0.002) | 0.022 | 1.69 (1.62, 1.76) | 0.91 (0.54, 1.56) | 1.02 (0.98, 1.06) | 0.284 |
|  |  |  |  |  |  |  |  |  |
| Type 2 diabetes | 0.003 (0.003, 0.004) | 0.002 (-0.005, 0.009) | 4 x10^-4^ (-2x10^-4^, 1x10^-4^) | 0.173 | 1.29 (1.22, 1.35) | 1.44 (0.88, 2.36) | 0.99 (0.95, 1.03) | 0.656 |
|  |  |  |  |  |  |  |  |  |
| Coronary heart disease | 0.007 (0.006, 0.008) | -0.002 (-0.012, 0.009) | 0.001 (-0.000, 0.002) | 0.261 | 1.28 (1.23, 1.32) | 1.58 (1.08, 2.31) | 0.99 (0.96, 1.03) | 0.657 |
|  |  |  |  |  |  |  |  |  |
| Stroke | 1x10^-4^ (-5x10^-4^, 8x10^-4^) | -0.003 (-0.009, 0.003) | 5x10^-5^ (-5x10^-4^, 6x10^-4^) | 0.856 | 1.02 (0.95, 1.08) | 0.86 (0.43, 1.72) | 1.00 (0.95, 1.06) | 0.887 |
|  |  |  |  |  |  |  |  |  |

# Table S13 – Effect modification of genetic predisposition to cardiovascular risk factors and disease by childhood maltreatment score on the additive and multiplicative scale at P<5x10^-8^, adjusted for covariate interactions.

# Interactions considered were those between main confounders (age, sex, and genetic principal components) and the polygenic scores or maltreatment scores. Analyses were also adjusted for age, sex and 40 genetic principal components. BMI=body mass index; LDL=low-density lipoprotein; PGS=polygenic score.

# Table S14 – Effect modification of genetic predisposition to cardiovascular risk factors and disease by exposure to childhood maltreatment on the additive and multiplicative scale at P<5x10^-8^, adjusted for childhood SEP.

|  | **Adjusted for childhood SEP, P<5x10^-8^** | | | | | | | |
| --- | --- | --- | --- | --- | --- | --- | --- | --- |
| **Continuous Trait** | **Additive scale** | | | | **Multiplicative scale** | | | |
|  | **Mean difference in SD of trait per SD increase of PGS (95% CI)** | **Mean difference in SD of trait per unit increase of maltreatment score (95% CI)** | **Interaction coefficient** | **P value of interaction** | **Mean difference in SD of log trait per SD increase of PGS (95% CI)** | **Mean difference in log SD of trait per unit increase of maltreatment score (95% CI)** | **Interaction coefficient** | **P value of interaction** |
| BMI | 0.12 (0.12, 0.13) | 0.057 (0.051, 0.063) | 0.010 (0.004, 0.016) | 0.002 | 0.12 (0.12, 0.13) | 0.056 (0.050, 0.062) | 0.009 (0.003, 0.015) | 0.006 |
| Drinks per week | 0.04 (0.04, 0.05) | 0.002 (-0.004, 0.008) | 4x10^-4^ (-6x10^-3^, 6x10^-3^) | 0.905 | 0.05 (0.04, 0.05) | -0.025 (-0.031, -0.019) | 0.001 (-0.005, 0.007) | 0.807 |
| LDL cholesterol | 0.23 (0.22, 0.24) | 2x10^-4^ (-6x10^-4^, 6x10^-4^) | 0.004 (-0.002, 0.010) | 0.160 | 0.23 (0.22, 0.23) | -0.001 (-0.007, 0.005) | 0.005 (-0.001, 0.011) | 0.091 |
| Lifetime smoking | 0.04 (0.03, 0.04) | 0.087 (0.081, 0.093) | 0.003 (-0.003, 0.010) | 0.269 | 0.04 (0.03, 0.04) | 0.090 (0.084, 0.096) | 0.004 (-0.003, 0.010) | 0.249 |
| Systolic blood pressure | 0.09 (0.09, 0.10) | -0.010 (-0.016, -0.004) | -0.001 (-0.007, 0.005) | 0.776 | 0.09 (0.09, 0.10) | -0.011 (-0.017, -0.005) | -0.001 (-0.007, 0.005) | 0.795 |
| **Binary Trait** | **Risk difference of trait per SD increase of PGS (95% CI)** | **Risk difference of trait per SD increase of maltreatment score (95% CI)** | **Interaction coefficient** | **P value of interaction** | **OR of trait per SD increase of PGS (95% CI)** | **OR of trait per unit increase of maltreatment score (95% CI)** | **Interaction coefficient** | **P value of interaction** |
| Atrial fibrillation | 0.011 (0.010, 0.012) | 0.001 (-0.000, 0.001) | 0.001 (-0.000, 0.001) | 0.227 | 1.72 (1.64, 1.79) | 1.01 (0.96, 1.06) | 1.03 (0.99, 1.07) | 0.183 |
|  |  |  |  |  |  |  |  |  |
| Type 2 diabetes | 0.003 (0.003, 0.004) | 0.002 (0.002, 0.003) | 0.001 (-0.000, 0.001) | 0.082 | 1.29 (1.22, 1.36) | 1.17 (1.12, 1.23) | 1.00 (0.96, 1.05) | 0.865 |
|  |  |  |  |  |  |  |  |  |
| Coronary heart disease | 0.007 (0.005, 0.008) | 0.003 (0.002, 0.004) | 0.001 (-0.001, 0.002) | 0.320 | 1.27 (1.22, 1.32) | 1.12 (1.08, 1.17) | 1.00 (0.97, 1.04) | 0.963 |
|  |  |  |  |  |  |  |  |  |
| Stroke | 6x10^-5^ (-6x10^-4^, 7x10^-4^) | 5x10^-4^ (-1x10^-4^, 1x10^-3^) | 2x10^-5^ (-6 x10^-4^, 6x10^-4^) | 0.956 | 1.01 (0.94, 1.08) | 1.05 (0.99, 1.12) | 1.00 (0.94, 1.07) | 0.970 |
|  |  |  |  |  |  |  |  |  |

# Proxies of childhood SEP utilised were number of siblings and maternal smoking. Analyses were also adjusted for age, sex and 40 genetic principal components. BMI=body mass index; LDL=low-density lipoprotein; PGS=polygenic score.

# References

1. Mitchell, R., Hemani, G., Dudding, T., Corbin, L., Harrison, S., Paternoster L. UK Biobank Genetic Data: MRC-IEU Quality Control, version 2 - Datasets - data.bris. DataBris. 2018;

2. Hemani G, Zheng J, Elsworth B, Wade KH, Haberland V, Baird D, et al. The MR-Base platform supports systematic causal inference across the human phenome. Loos R, editor. Elife. 2018;7:e34408.

3. Choi SW, Mak TSH, O’Reilly PF. Tutorial: a guide to performing polygenic risk score analyses. Nat Protoc. 2020;15(9):2759–72.

4. Carter AR, Gill D, Davies NM, Taylor AE, Tillmann T, Vaucher J, et al. Understanding the consequences of education inequality on cardiovascular disease: Mendelian randomisation study. The BMJ. 2019;365:1–12.

5. Locke AE, Kahali B, Berndt SI, Justice AE, Pers TH, Day FR, et al. Genetic studies of body mass index yield new insights for obesity biology. Nature. 2015;518(7538):197–206.

6. Howe LJ, Lawson DJ, Davies NM, St. Pourcain B, Lewis SJ, Davey Smith G, et al. Genetic evidence for assortative mating on alcohol consumption in the UK Biobank. Nat Commun. 2019;10(1).

7. Liu M, Jiang Y, Wedow R, Li Y, Brazel DM, Chen F, et al. Association studies of up to 1.2 million individuals yield new insights into the genetic etiology of tobacco and alcohol use. Nat Genet. 2019;51(2):237–44.

8. UK Biobank. Biomarker assay quality procedures: approaches used to minimise systematic and random errors (and the wider epidemiological implications). 2019;(April):25.

9. Willer CJ, Schmidt EM, Sengupta S, Peloso GM, Gustafsson S, Kanoni S, et al. Discovery and refinement of loci associated with lipid levels. Nat Genet. 2013;45(11):1274–85.

10. Wootton RE, Richmond RC, Stuijfzand BG, Lawn RB, Sallis HM, Taylor GMJ, et al. Evidence for causal effects of lifetime smoking on risk for depression and schizophrenia: A Mendelian randomisation study. Psychol Med. 2020;50(14):2435–43.

11. Ellinor PT, Lunetta KL, Albert CM, Glazer NL, Ritchie MD, Smith A V., et al. Meta-analysis identifies six new susceptibility loci for atrial fibrillation. Nat Genet. 2012;44(6):670–5.

12. Scott RA, Scott LJ, Mägi R, Marullo L, Gaulton KJ, Kaakinen M, et al. An Expanded Genome-Wide Association Study of Type 2 Diabetes in Europeans. Diabetes. 2017;66(11):2888–902.

13. Nikpay M, Goel A, Won HH, Hall LM, Willenborg C, Kanoni S, et al. A comprehensive 1000 Genomes-based genome-wide association meta-analysis of coronary artery disease. Nat Genet. 2015;47(10):1121–30.

14. Malik R, Chauhan G, Traylor M, Sargurupremraj M, Okada Y, Mishra A, et al. Multiancestry genome-wide association study of 520,000 subjects identifies 32 loci associated with stroke and stroke subtypes. Nat Genet. 2018;50(4):524–37.

15. Millwood IY, Walters RG, Mei XW, Guo Y, Yang L, Bian Z, et al. Conventional and genetic evidence on alcohol and vascular disease aetiology: a prospective study of 500 000 men and women in China. The Lancet. 2019 May 4;393(10183):1831–42.

16. Larsson SC, Bäck M, Rees JMB, Mason AM, Burgess S. Body mass index and body composition in relation to 14 cardiovascular conditions in  UK Biobank: a Mendelian randomization study. Eur Heart J. 2020 Jan;41(2):221–6.

17. Ference BA, Ginsberg HN, Graham I, Ray KK, Packard CJ, Bruckert E, et al. Low-density lipoproteins cause atherosclerotic cardiovascular disease. 1. Evidence  from genetic, epidemiologic, and clinical studies. A consensus statement from the European Atherosclerosis Society Consensus Panel. Eur Heart J. 2017 Aug;38(32):2459–72.

18. Cheung BMY, Lauder IJ, Lau CP, Kumana CR. Meta-analysis of large randomized controlled trials to evaluate the impact of statins on cardiovascular outcomes. Br J Clin Pharmacol. 2004 May;57(5):640–51.

19. Larsson SC, Mason AM, Bäck M, Klarin D, Damrauer SM, Program MV, et al. Genetic predisposition to smoking in relation to 14 cardiovascular diseases. Eur Heart J. 2020 Sep 14;41(35):3304–10.

20. Ettehad D, Emdin CA, Kiran A, Anderson SG, Callender T, Emberson J, et al. Blood pressure lowering for prevention of cardiovascular disease and death: a systematic review and meta-analysis. The Lancet. 2016 Mar 5;387(10022):957–67.

21. Ahmad OS, Morris JA, Mujammami M, Forgetta V, Leong A, Li R, et al. A Mendelian randomization study of the effect of type-2 diabetes on coronary heart  disease. Nat Commun. 2015 May;6:7060.
