# Supplementary material for: Investigating effect modification between childhood maltreatment and genetic risk for cardiovascular disease in the UK Biobank
Source: PLoS One. 2023 May 4;18(5):e0285258. doi: 10.1371/journal.pone.0285258 (PMC10159177; doi:10.1371/journal.pone.0285258)
Supplement: Attachment — Submitted filename: Response to reviewers.docx [file pone.0285258.s003.docx]

# Editorial comments:

We would like to thank all Editors and Reviewers for their careful and considered review of our work. We are pleased to have had the opportunity to respond to all comments below.

All editor/reviewer comments are in blue font, with our responses in black font. We have included relevant sections of text in *italics* and highlighted any additions in ***bold underlined font.***

## Comment 1: Please ensure that your manuscript meets PLOS ONE's style requirements, including those for file naming. The PLOS ONE style templates can be found at  <https://journals.plos.org/plosone/s/file?id=wjVg/PLOSOne_formatting_sample_main_body.pdf> and  <https://journals.plos.org/plosone/s/file?id=ba62/PLOSOne_formatting_sample_title_authors_affiliations.pdf>

Response: We have read the relevant templates and confirm that our manuscript and supplementary material meet these formatting requirements. The “Funding information”, “Author contributions” and “Disclosures” sections were removed and section header format was altered to match the guidelines specified in https://journals.plos.org/plosone/s/submission-guidelines.

## Comment 2: We note that the grant information you provided in the ‘Funding Information’ and ‘Financial Disclosure’ sections do not match. When you resubmit, please ensure that you provide the correct grant numbers for the awards you received for your study in the ‘Funding Information’ section.

Response: We thank the editors for noticing this mismatch. We can confirm we have checked the two sections and the funding information provided in the submission process is correct. We have removed the funding information section from the manuscript file as explained above.

## Comment 3: We note that you have stated that you will provide repository information for your data at acceptance. Should your manuscript be accepted for publication, we will hold it until you provide the relevant accession numbers or DOIs necessary to access your data. If you wish to make changes to your Data Availability statement, please describe these changes in your cover letter and we will update your Data Availability statement to reflect the information you provide.

Response: We can confirm we have checked the data availability statement and these details are correct.

## Comment 4: Please include your full ethics statement in the ‘Methods’ section of your manuscript file. In your statement, please include the full name of the IRB or ethics committee who approved or waived your study, as well as whether or not you obtained informed written or verbal consent. If consent was waived for your study, please include this information in your statement as well.

Response: We have now added the full ethics statement that was provided in the submission process under the ‘Methods’ section, reading the following:

***“UKB has approval from the North West Multi-centre Research Ethics Committee (MREC) as a Research Tissue Bank (RTB) approval. All analyses were performed under approved UKB project 19278. As part of the UKB recruitment process, all necessary patient/participant consent was obtained and the appropriate institutional forms were archived. Data were analyzed anonymously.”***

## Comment 5: Please include captions for your Supporting Information files at the end of your manuscript, and update any in-text citations to match accordingly. Please see our Supporting Information guidelines for more information: <http://journals.plos.org/plosone/s/supporting-information>.

Response: We can confirm captions for the Supplementary Information files are now included at the end of the main text, after the ‘References’ section.

Comment 6: Please review your reference list to ensure that it is complete and correct. If you have cited papers that have been retracted, please include the rationale for doing so in the manuscript text, or remove these references and replace them with relevant current references. Any changes to the reference list should be mentioned in the rebuttal letter that accompanies your revised manuscript. If you need to cite a retracted article, indicate the article’s retracted status in the References list and also include a citation and full reference for the retraction notice.

Response: We can confirm all references are complete and correct. No citations have been retracted.

Reviewers' comments:

# Reviewer 1:

## This is a great paper though there was no finding on significant interaction between childhood maltreatment and PGS influencing CVD.

We thank the reviewer for noting the importance of this paper. We agree that whilst the findings were largely null, this is still important to report.

## Comment 1: There may be no interaction indeed, however, a true association may have not been revealed due to low statistical power using the 1 df test of the interaction effect alone. The 2 df test is more powerful when PGS is weak, and the interaction effect is moderate than either the 1df test of the PGS or the 1df test of the interaction alone. Using the 2df joint test under the H0: Beta(PGS) = Beta(PGX x childhood maltreatment), you may be able to identify associations that were not discovered using the 1df test.

Response: We agree with the reviewer that this is a very important point; we may not have found evidence of an interaction due to a lack of statistical power. We have included a comment on this in the discussion in line 277:

*“****Despite UK Biobank being the largest available study with data on both childhood maltreatment and genetic data (required to derive polygenic scores), it is possible our analyses were still underpowered to detect evidence of effect modification.*** *Therefore, replication and validation using other cohorts if they become available, may elucidate whether we missed evidence of effect modification and/or sex-specific effects due to a lack of statistical power.”*

We thank the reviewers for the suggestion of using a 2df joint test, but we respectfully disagree that this method would be appropriate for our analyses.

The 2df method, also known as joint hypothesis testing, tests a joint null hypothesis relating to both the main genetic effect and the interaction term (H0: Beta(PGS) = Beta(PGX x childhood maltreatment) = 0). This is an approach to jointly test genetic effects and potential G*E interactions i.e. testing genetic effect whilst allowing for interaction (PMID: 18538979).

As mentioned by the reviewer, this method is more powerful for detecting genetic effects, and has been extensively used to detect genetic variants (e.g. 10.1016/j.ajhg.2018.01.015), but is not appropriate to our research question as we aim to detect and estimate G*E interactions. The null hypothesis in the 2df join test is rejected when either or both of these coefficients is are not 0. We would generally expect to be the case as the polygenic scores have an association with their respective traits and thus would detect an effect whilst giving us no insight into whether there was a G*E effect.

## Comment 2: Have you considered including age squared as one of the covariates? In case including age only in the model would not be sufficient to control for the effect of age.

Response: We thank the reviewer for noting this. In our findings, the adjustment for a linear age term did not strongly attenuate or alter the direction of any effect estimates, including our BMI*maltreatment findings. We therefore feel there may be limited value to including an additional age term.

## Comment 3: A minor suggestion is that I found some typos or grammatical errors in the context. For example, in the method for effect modification, I was wondering whether you meant multivariable “logistic” regression for binary traits, not “linear” regression.

Response: We thank the reviewer for noticing these errors, and we have completed a full spelling and grammar check of the manuscript.

When we considered binary outcomes on the additive scale, we conducted analyses using linear regression to estimate a risk difference effect. We have checked all instances of logistic and linear regression to ensure each time they refer to the correct analysis conducted.

# Reviewer 2:

## Comment 1: Statistical significance of the interaction (p-value) should be mentioned in the abstract.

Response: In line with guidance from the American Statistical Association we have avoided using P values to dichotomise results into significant or non-significant. However, we appreciate these can sometimes be helpful to a reader to interpret the results. We have now added the P value for the interaction term for BMI PGS*maltreatment in relation to BMI into the abstract:

*“On the additive scale, childhood maltreatment accentuated the effect of genetic susceptibility to higher BMI* ***(P_effect modification_: 0.003)****. Individuals not exposed to childhood maltreatment had an increase in BMI of 0.12 SD (95% CI: 0.11, 0.13) per SD increase in BMI PGS, compared to 0.17 SD (95% CI: 0.14, 0.19) in those exposed to all types of childhood maltreatment.”*

## Comment 2: Table 2 presented the variable distributions in the analytical sample versus the whole UKB to show that the distributions of various factors were not substantially different. This information is better placed into supplementary materials, since the information of the whole UKB was not directly related to this study and is very likely available from other studies that used the full cohort. Instead, showing the sample characteristics by the childhood maltreatment score or by sex would be more informative.

Response: We thank the reviewer for noting this, and for the helpful suggestion. In the interest of the legibility of the table, we have presented the study sample characteristics according to sex. This information is now presented in Table 2. We changed the text corresponding to the table to reflect some of the information conveyed by stratification according to sex:

*“Eligible participants had a mean age at baseline of 56 years (SD: 8.0 years) and 57% were female.* ***Overall maltreatment scores did not differ largely between sexes, yet more female participants reported sexual and emotional abuse and less physical abuse compared to male participants (Table 2). Male participants had slightly higher rates of CVD incidence.***”

We have since moved the original table to the supplementary material as Table S5.

## Comment 3: While it makes sense to analyze the continuous traits as per SD difference, the results would be more interpretable if the SD can be converted to the actual units (e.g., how much is per SD of BMI in this analytical sample?)

Response: We agree this is an important point made by the reviewer. The SD of all continuous variables are presented in the Sample Characteristics table (Table S5). However, as this is an important point for the interpretation of the results, we have additionally stated the SD of BMI in line 191, where we first present results of BMI:

*“ Childhood maltreatment score was associated with higher BMI (β: 0.07 SD; 95% CI: 0.06, 0.07)****, where 1 SD corresponds to 4.38 kg/m^2^,*** *and lifetime smoking, as well as with T2DM and CHD incidence (Table S5).”*

## Comment 4: Childhood maltreatment score was used as a continuous variable in all analyses. The underlying hypothesis is that different types of maltreatment have the same equal effect on cardiovascular traits, which may not be true. The authors did not examine potential heterogeneity in the associations by maltreatment types although the data are available (e.g., sexual abuse is often considered the more severe form of childhood maltreatment).

Response: The potential differences between the types of maltreatment and their effects on CVD are very important considerations and we thank the reviewer for pointing this out.

Our previous work in the same cohort has shown that there is limited heterogeneity in the associations between CVD and different types of maltreatment (10.1136/heartjnl-2019-316320). We therefore did not repeat those comparisons in this work. To reflect this in the text, we have added the following text under the Discussion section (line 273):

*“Integrating the five types of childhood maltreatment into a score, while relevant in the light of dose-response relationships between childhood maltreatment and CVD, may miss maltreatment type-specific effects.* ***Nonetheless, previous work in this cohort has shown there is limited heterogeneity in the associations with CVD across maltreatment types [13]****.”*

We agree with the importance of considering the differing severities each type of maltreatment. The maltreatment score used in our work, calculated according to the Childhood Trauma Screener, partly addresses the different severities of these exposures by having different cut-offs according to the type of maltreatment. This information is detailed in Table S1 and the following text was edited under the Methods section (line 79):

*“The types of maltreatment assessed comprised of emotional neglect, physical neglect, emotional abuse, physical abuse and sexual abuse [25,26]. A summary score of childhood maltreatment was calculated as per the Childhood Trauma Screener [25]****. The score reflects*** *the cumulative number of maltreatment types experienced by an individual, spanning from 0 to 5****, according to cut-offs specific to each type*** *(Table S1).”*

We however agree that despite the inclusion of these cut-offs, the maltreatment score does not completely address potential differences between maltreatment types, as discussed in line 266, or capture other important aspects of these exposures, such as the ones highlighted by the reviewer in Comment 6. We therefore have added greater discussion of this limitation, as detailed under our response to Comment 6.

## Comment 5: The study only found a significant interaction between childhood maltreatment and genetic susceptibility to BMI. A recent study reported that BMI was the most important factor that mediated the association between childhood abuse and metabolic profiles in midlife (PMID: 35471987). Incorporating this study in the discussion may potentially increase the significance of the findings from the current study.

Response: We thank the reviewer for highlighting this study and agree it could be an important point to make. We have now included this in the discussion (line 257):

*“In a study with 161 individuals with major depression, Opel and colleagues also found childhood maltreatment modified polygenic susceptibility to BMI but in the opposite direction, with exposure to maltreatment reducing the effect of polygenic susceptibility on BMI [39]. These opposing findings could be due to different definitions of maltreatment (binary versus continuous score), or different study populations (depressive patients versus population cohort).* ***Contrastingly, a recent study found midlife BMI to be the major mediator in the association between early life abuse and midlife metabolomic profile [40]. These results, despite being specific to women, coupled with our findings, are in support of a potential role of childhood maltreatment in adverse metabolic outcomes in later life.****”*

## Comment 6: Discussion: it should be acknowledged that the assessment of childhood maltreatment was also crude that did not capture frequency, duration, severity or timing for each type of maltreatment. For example, childhood maltreatment that occurs earlier in childhood may result in stronger gene-environment interaction effect. Also, the definition of ‘child’ in the assessment question is vague. Someone could consider an “adolescent” as a child while others may not. The maltreatment during adolescence may not have the same biological effect as the one during early childhood <5 years old.

Response: We think this is an important point highlighted by the reviewer. A great strength of UK Biobank is the large sample size available for this analysis, however, the information available is not detailed enough to capture these potentially important aspects. To further acknowledge this important point we have included greater discussion of this as a limitation (line 339):

*“However, only a fraction of maltreatment cases is reported [52] and thus many cases might be missed.* ***As with most large studies, the measures used to construct the summary score were limited to type of maltreatment and self-reported frequency and thus do not capture aspects which may affect the severity of these exposures and their effect on health over the lifecourse, such as their timing in childhood, severity or duration.****”*
